# Image-based discrimination of the early stages of mesenchymal stem cell differentiation

**DOI:** 10.1091/mbc.E24-02-0095

**Published:** 2024-07-11

**Authors:** Justin Hoffman, Shiyuan Zheng, Huaiying Zhang, Robert F. Murphy, Kris Noel Dahl

**Affiliations:** a Department of Computational Biology, School of Computer Science, Carnegie Mellon University, Pittsburgh, PA 15213; b Department of Biomedical Engineering, College of Engineering, Carnegie Mellon University, Pittsburgh, PA 15213; c Department of Biological Sciences, Mellon College of Science, Carnegie Mellon University, Pittsburgh, PA 15213; d Department of Computational Biology, School of Computer Science, Carnegie Mellon University, Pittsburgh, PA 15213; e Department of Biomedical Engineering, College of Engineering, Carnegie Mellon University, Pittsburgh, PA 15213; Ben-Gurion University of the Negev

## Abstract

Mesenchymal stem cells (MSCs) are self-renewing, multipotent cells, which can be used in cellular and tissue therapeutics. MSCs cell number can be expanded in vitro, but premature differentiation results in reduced cell number and compromised therapeutic efficacies. Current techniques fail to discriminate the “stem-like” population from early stages (12 h) of differentiated MSC population. Here, we imaged nuclear structure and actin architecture using immunofluorescence and used deep learning–based computer vision technology to discriminate the early stages (6–12 h) of MSC differentiation. Convolutional neural network models trained by nucleus and actin images have high accuracy in reporting MSC differentiation; nuclear images alone can identify early stages of differentiation. Concurrently, we show that chromatin fluidity and heterochromatin levels or localization change during early MSC differentiation. This study quantifies changes in cell architecture during early MSC differentiation and describes a novel image-based diagnostic tool that could be widely used in MSC culture, expansion and utilization.

## Abstract (executive summary)

Differentiation of MSCs affects therapeutic efficacies.

Combining light microscope imaging and deep learning–based computer vision technology, the authors find that changes in nuclear structure can be detected during the early stage (first 6- to 24-h after differentiation) of adipogenesis.

The findings enhance our understanding of MSC differentiation and the approaches could expand the use of MSCs in stem cell therapies and tissue engineering.

## INTRODUCTION

Mesenchymal stem cells (MSCs) are self-renewing, multipotent stem cells of wide interest since their discovery from bone marrow in 1973 (Sonowal *et al.*, 2013). MSCs are currently therapeutically used both for immunoregulation and for their differentiation capacity. MSCs secrete cytokines that regulate innate and adaptive immune cells, and MSCs are used clinically for graft versus host disease (Amorin *et al.*, 2014; Fan *et al.*, 2020). Under well-defined differentiation conditions, multipotent MSCs differentiate to mesenchymal lineages including bone, adipose, cartilage, myelo-supportive stroma, smooth muscle, cardiomyocytes, and tendon (Wei *et al.*, 2013). Differentiated cells show phenotypes of native cells including secreting extracellular matrix, which can be used for tissue engineering. More recently, MSCs have shown potential to differentiate into ectodermal lineages such as neurons and endodermal lineages such as hepatocytes (Ong, Dai and Leong, 2006; Anghileri *et al.*, 2008).

MSC technologies require expansions of cells in culture for numerous cell generations in a non-differentiated stem-like state. Culture can take weeks to months, and it is essential to monitor stemness or cells may differentiate to random lineages, which slows proliferation and produces a heterogeneous product. Current phenotyping strategies rely on quantifying surface markers of stemness and markers of differentiation as well as gross changes in cell shape. The criteria for determining MSC stemness are generally grouped into three categories (Dominici *et al.*, 2006):

1. Morphological characteristics: MSCs are adherent and of consistent size and shape, typically spindle shaped and elongated. Cells change shape during differentiation, and manipulation of cell shape can influence the cell fate commitment (McBeath *et al.*, 2004). For example, osteogenic MSCs spread and flatten while adipogenesis is associated with round cells (Kilian *et al.*, 2010). These changes in cell shape have been correlated with changes in cytoskeletal architecture and tension (Yourek *et al.*, 2007; van Vliet and Agostinis, 2017).
2. Surface markers: MSC show the presence of stemness surface markers including CD105, CD73, CD90 and updated stemness markers like stage-specific embryonic antigen (SSEA)-1, SSEA-4, CD271, CD146, Stro-1, while negative expression of CD45, CD34, CD14, CD11b, CD79α, CD19, and HLA-DR. The list of stem cell–specific markers is being continuously refined and often reflects a change in levels rather than binary results.
3. Differentiation ability: MSCs have osteogenic, adipogenic, chondrogenic differentiation capacity in vitro. This is achieved by 2–3 wk exposure to differentiation media and measurements of new surface markers and production of extracellular matrix (Solchaga, Penick and Welter, 2011; Munir *et al.*, 2017; Hanna, Mir and Andre, 2018).

Morphological characteristics and expressed “stemness” markers fail to discriminate true multipotent MSC cells from lineage-transitioning (early differentiating) cells and continued populations. For example, expression of markers during adipogenesis (PPARγ) and during osteogenesis (RUNX2) are comparable in the early stages of differentiation stages (Aldridge *et al.*, 2013; Gromolak *et al.*, 2020; Robert *et al.*, 2020). Thus, known surface markers and morphological markers can only measure late-differentiating cells (after 2–3 wk differentiation) (Solchaga, Penick and Welter, 2011; Munir *et al.*, 2017; Ghaneialvar *et al.*, 2018; Hanna, Mir and Andre, 2018). Surface markers have an additional shortcoming: cells typically need to be suspended, have immunoglobulins bound to their surface and be imaged by flow cytometry. To achieve this, either a subset of cells is measured and results are extrapolated to the population or cells are manipulated and then added back to culture.

We aimed to develop a methodology based solely on imaging that could eventually be used by standard light microscopy to identify stemness and early differentiation. We hypothesized that MSC morphology information could be effective in determining cell phenotype since MSC differentiation is associated with changes in actin filament organization as well as nuclear stiffness and chromatin mobility (Maloney *et al.*, 2010; Nava *et al.*, 2020). Actin images have recently been used to model myoblast differentiation (Shakarchy *et al.,* 2024). Also, mechanical and structural cues are involved in the maintenance of stemness of MSCs, lineage-specific differentiation and intracellular trafficking (Petzold and Gentleman, 2021).

We used standard widefield fluorescence microscopy to image MSC structures (actin and chromatin) at various states of early differentiation and applied computer vision methods to distinguish those states (see Figure 5 in *Materials and Methods*). Our results demonstrate that the differentiation state was accurately predicted using chromatin images. We expect our approach to be useful for practical monitoring of MSC differentiation state.

## RESULTS

### Cell structure changes during adipogenic differentiation

**FIGURE 1:**
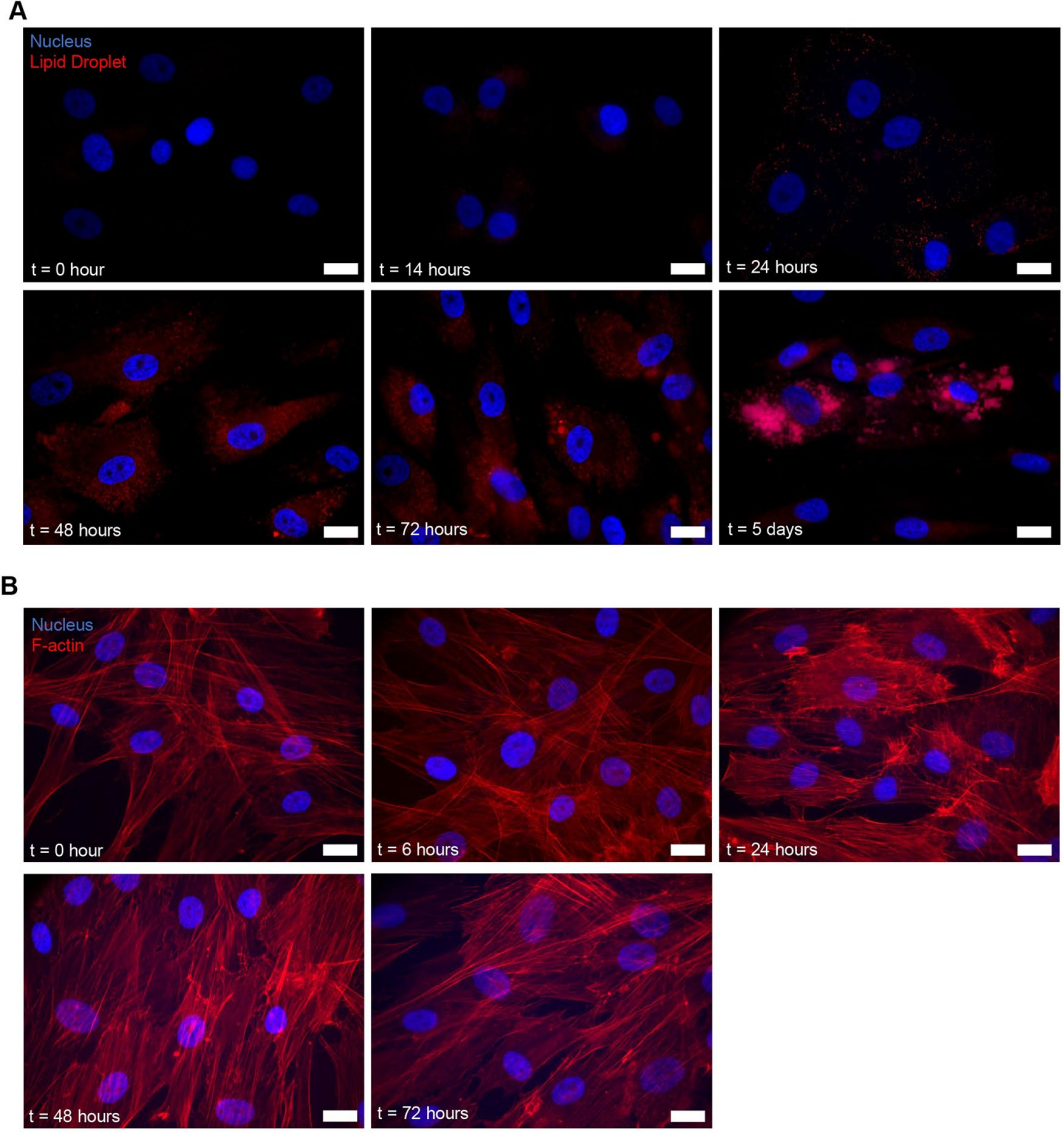
Morphological markers lipid droplet and cytoskeleton were stained with Nile red and rhodamine phalloidin, respectively. (A) Lipid droplets were stained with Nile red (red) and nuclei were stained with Hoechst 33342 (blue) at different timepoints during MSC adipogenesis. (B) F-actin was stained with rhodamine phalloidin (red) and nuclei were stained with Hoechst 33342 (blue) at different timepoints during MSC adipogenesis. The scale bar is 20 μm.

To provide a baseline for our computer vision studies, we first examined a number of markers that have been used previously to characterize MSC differentiation. We began by imaging lipid droplets, a key morphological marker during adipogenesis (using Nile red) and F-actin (using phalloidin). As seen in Figure 1A, lipid droplets were detectable 48 h after the initiation of adipogenic differentiation (referred to here as 48-h adipo), but not at 14-h adipo. Imaging F-actin (Figure 1B) revealed wispy F-actin fibers distributed along the long axis of the cell at 0 and 6 h. Beginning at 24-h adipo, we observed a remodeled, denser actin cytoskeleton, consistent with previous reports with imaging and mechanical measurements (Maloney *et al.*, 2010). We conclude that visual examination of these markers is not sufficient to measure early changes.

**FIGURE 2:**
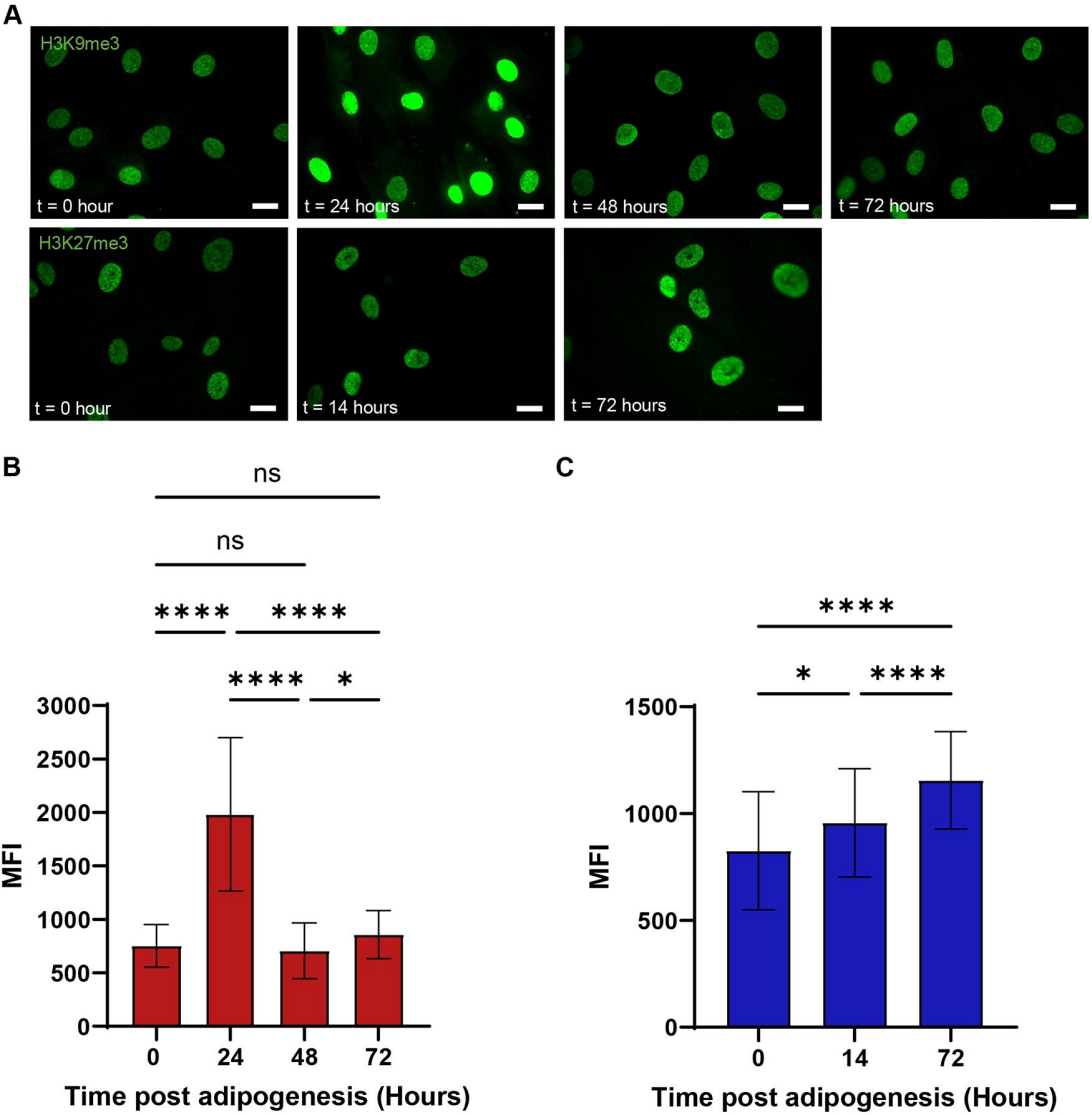
Heterochromatin markers H3K9me3 and H3K27me3 were assessed with widefield immunocytochemistry. (A) Example immunostaining images of heterochromatin markers H3K9me3 (first row, green) and H3K27me3 (second row, green) in MSC adipogenesis at different timepoints. The scale bar is 20 μm. (B) Quantification of mean fluorescent intensity (MFI) for H3K9me3 stained cells (*n* = 62). Statistical analysis was performed with the Kruskal–Wallis test. ns, not significant; *, *P* < 0.05; **, *P* < 0.01; ***, *P* < 0.001; ****, *P* < 0.0001. (C) Quantification of MFI from H3K27me3 stained cells (*n* = 62). Statistical analysis was performed with one-way ANOVA, *P* > 0.05, ns; *, *P* < 0.05; **, *P* < 0.01; ***, *P* < 0.001; ****, *P* < 0.0001.

We also imaged heterochromatin during adipogenic differentiation using immunocytochemistry since changes in heterochromatin associated with gene expression changes are expected for stem cell differentiation. It has been shown that heterochromatin levels change dramatically during embryonic stem cell differentiation, and there are gene-specific changes in heterochromatin in single-cell MSC differentiation (Dahl *et al.*, 2006; Nicetto *et al.*, 2019). We labeled cells with heterochromatin markers H3K9me3 and H3K27me3 at different timepoints after initiation of adipocyte differentiation. As shown in Figure 2A, heterochromatin was evenly distributed within the cell nucleus in MSCs during early adipogenic differentiation. Previous studies showed that H3K9me3 and H3K27me3 localization was different after 7-day differentiation (Stachecka *et al.*, 2021). Traditional image analysis of widefield images (see *Materials and Methods*) was performed to approximate levels of heterochromatin using these markers. H3K9me3 showed increased expression at 24-h adipo and then returned to initial levels (Figure 2B). H3K27me3 showed a gradual increase over 72 h (Figure 2C), consistent with previous studies of 7-day adipo (Stachecka *et al.*, 2021).

### Chromatin dynamics during adipogenic differentiation

**FIGURE 3:**
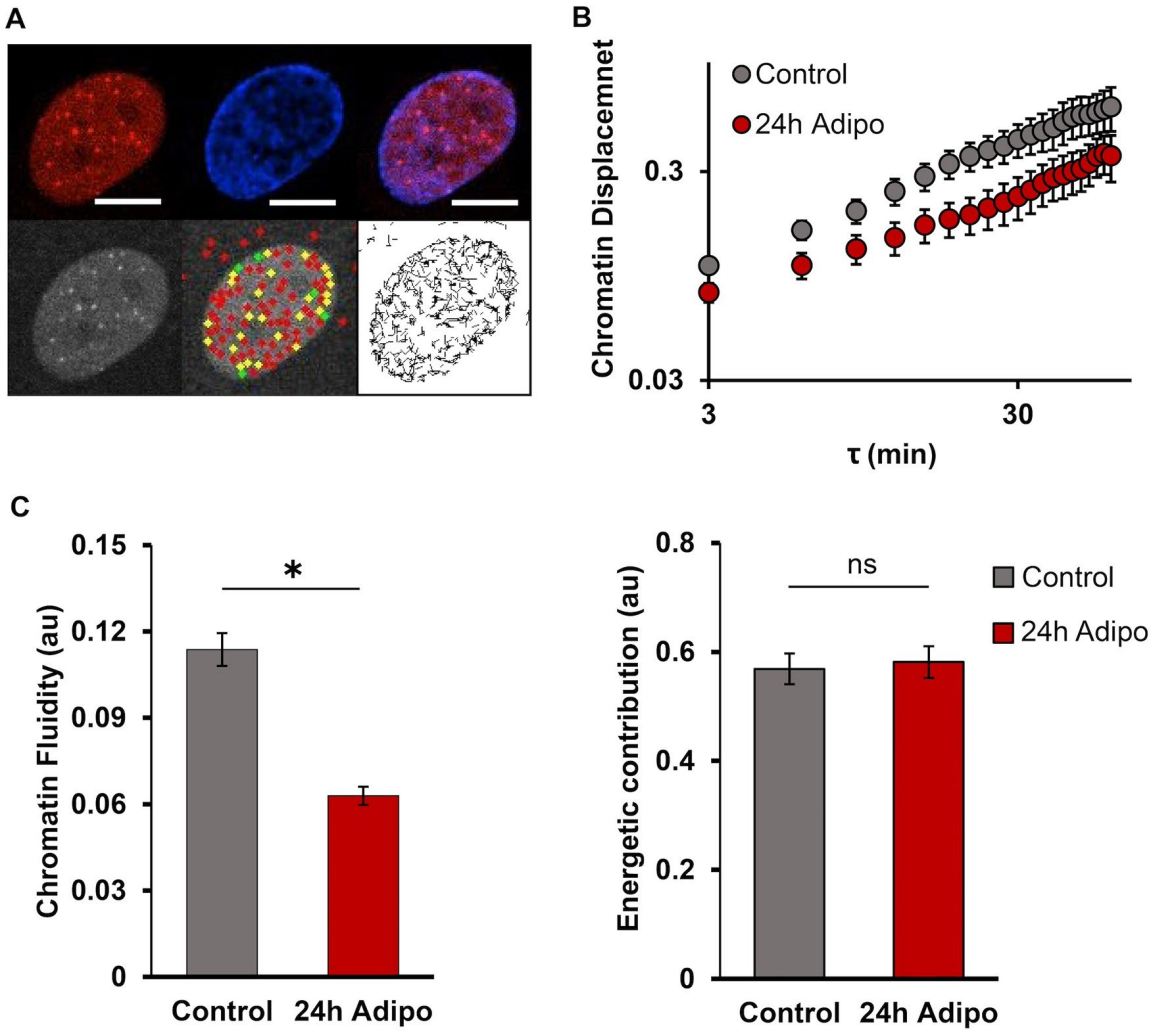
Chromatin mobility was decreased at 24-h after adipogenic differentiation. (A) Representative images of cell nuclei expressing mCherry2-TRF1. From left to right, the top row represents the mCherry, Hoechst, and merged channels, respectively. The bottom row represents the removal of nucleus movement, the possibility map of “real” puncta within the cell nucleus (Red, yellow, and green represent the least, intermediate, and highest possibility, respectively) and the trajectory of puncta movements over time. (B) Comparisons of CD for control (Time = 0 h) and 24-h adipo are displayed on a log-log scale. *N* = 12. (C) Parameters associated with chromatin fluidity decrease are shown for control and 24-h adipo (reduced fluidity is assessed from chromatin mobility). Parameters for force generated by the actin-myosin were statistically similar between control and 24-h adipo. ns, not significant; *, *P*< 0.05.

Our findings indicate that heterochromatin markers H3K9me3 and H3K27me3 differ in expression profiles during 72-h adipogenesis. H3K9me3 is significantly upregulated 24-h adipo, followed by a subsequent decrease to a similar level of 0-h adipo. H3K27me3, however, increases across time and reaches the highest level at 72-h adipo, which is consistent with previous studies (Stachecka *et al.*, 2021). Additionally, our work and that of others have shown that nuclear deformability changes during phenotypic changes including differentiation (Spagnol *et al.*, 2014; Heo *et al.*, 2016; Nava *et al.*, 2020). Given that the repressive histone markers greatly increase in early stage of differentiation and nucleus becomes more deformable, we further investigated the changes in chromatin mobility. We utilized particle tracking and image analysis to process the trajectories of DNA-binding probe mCherry2-TRF1 (see *Materials and Methods*). Chromatin displacement (CD) significantly decreased 24-h adipo compared with the undifferentiated control (Figure 3A). Applying our previously described methods for interpreting the trajectories (Spagnol and Dahl, 2014; Tedesco *et al.*, 2022), we determined that chromatin fluidity decreased at 24 h (Figure 3B). Less fluid chromatin at 24 h may correlate with the downregulation in proliferation and cell cycle–associated genes (Hung *et al.*, 2004; Marcon *et al.*, 2019). Together, it appears that mechanical properties within the nucleus change; unfortunately quantitative correlations are not available from static images since there is no obvious single protein marker (or none currently identified) that changes during early adipogenic differentiation.

### Image-based distinction between phases of early MSC differentiation

Because there are changes in MSC structure with differentiation, and these changes are not obvious with standard imaging, we examined whether deep learning-based computer vision methods could identify differentiation. For this, widefield fluorescent images of the actin cytoskeleton and nucleus, taken at 0, 6, 24, 48, and 72 h after initiating differentiation, were automatically segmented into single nuclei regions and used to produce four image collections: nucleus-only (Hoechst 33342–labeled DNA), actin only (rhodamine phalloidin–labeled F-actin), nucleus and actin, and bright field only. Models were trained using deep learning to identify image features that could distinguish between the different treatment times.

**FIGURE 4:**
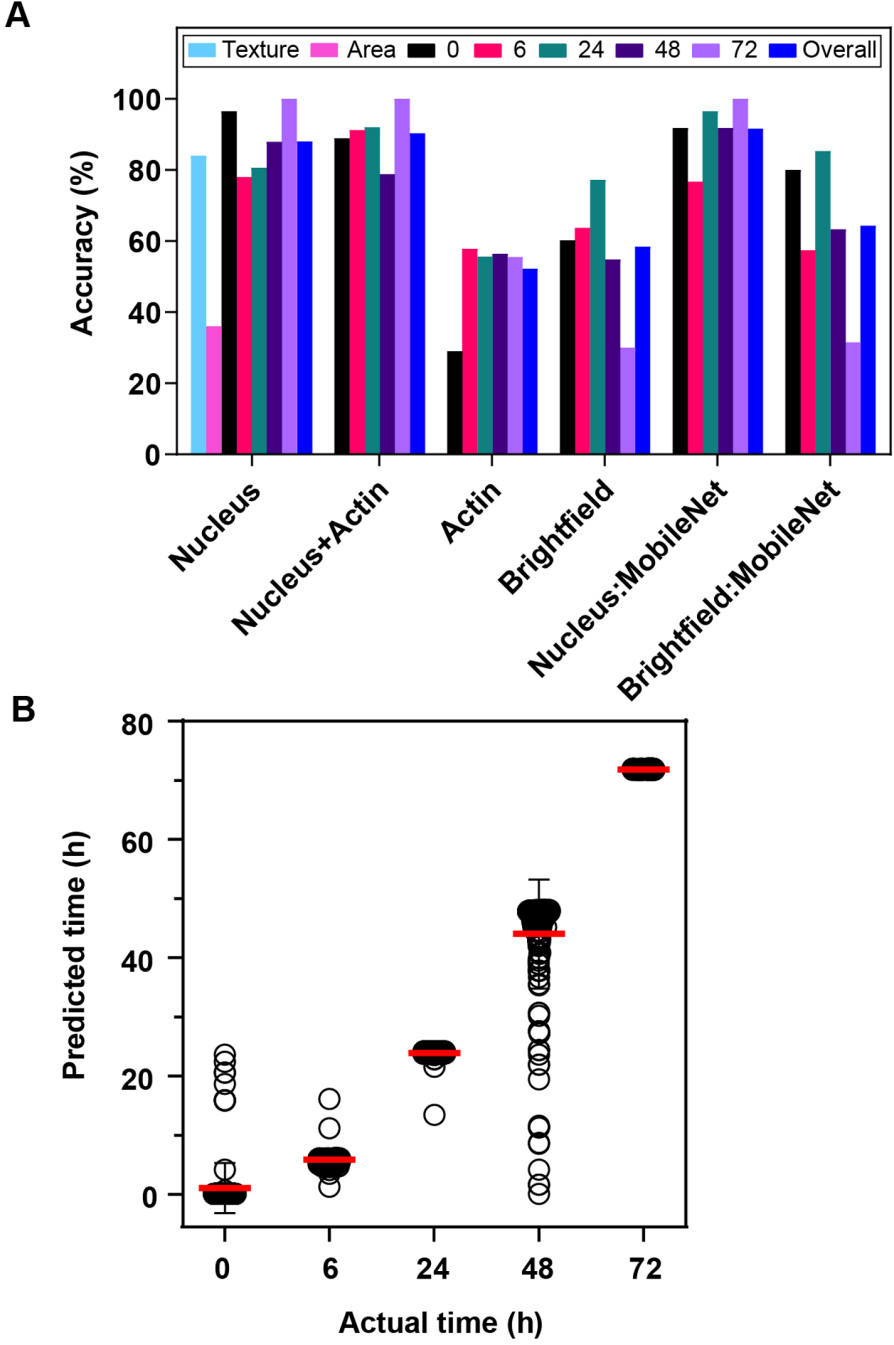
Detection of MSC differentiation stages using deep learning classification and regression. (A) Classification models were trained to predict the timepoints of segmented cell images in the training dataset, and the accuracy measured for the test set. Nuclear images are more informative than actin images for training a small network with low learnable parameters. Combining actin and nuclei provides a small accuracy boost of ∼2%. Brightfield images do significantly better than random (20%) but not as well as nuclear images. Larger models with more learnable parameters (MobileNet) perform similarly to small networks. Results are also shown for traditional image analysis using nuclear area or texture. (B) Using just the nucleus dataset, a regression model was trained to predict the time post differentiation using the training set, and treatment time was predicted for each nucleus in the test set. Results are for 271-462 cells per timepoint (see *Materials and Methods*).

Each dataset was divided into training and testing sets (all segmented regions from the same widefield image were kept in the same set), and a compact convolutional neural network (CNN) model was trained on the segmented regions to predict which of the five different time intervals they were from. Figure 4A shows the accuracy for the testing folds of the four collections. The nucleus-only collection showed 88% accuracy for the testing data. Including the actin information (both nucleus and actin) resulted in a 2% increase in accuracy to 90%. Images that contained actin only showed only a 52% accuracy, using a larger network architecture, such as MobileNet, provided relatively minimal performance boost. Images containing bright field alone achieved 58% accuracy with the small CNN and 64% accuracy with MobileNet. Note that with five roughly equal classes, the expected accuracy for a random classifier is 20%. Thus, even bright field alone provides accuracy significantly better than random, and the nucleus plus actin images provide excellent ability to distinguish differentiation states.

A natural question is whether similar results could be obtained with more traditional image analysis methods. For example, Haralick texture features (Haralick *et al.*, 1973) have been widely used to distinguish patterns, including subcellular patterns (Boland *et al.*, 1998). Calculating those on the nuclear images alone and training a random forest classifier achieved an overall accuracy of 84%, only 6% less than the deep learning approach. Using nuclear area was not sufficient, achieving an accuracy of only 36%.

These results demonstrate that there are significant changes in the spatial patterning of chromatin in the nucleus as differentiation proceeds. However, we cannot distinguish whether distinct changes are occurring at each timepoint or whether the timepoints are distinguishable because of the extent of a single change (or a small number of changes). To further explore these differences, we used a regression approach in place of classification. In this case, the learner seeks not to predict the class (timepoint) of each image but rather to predict the timepoint value itself. As shown in Figure 4B, a regression CNN is able to accurately estimate the length of time since a given nucleus began differentiation.

It is worth noting that all cells in a given population may not differentiate at exactly the same rate. This may explain the very small fraction of cells that are not classified or predicted “correctly” for a given timepoint. The regression model can predict each cell’s “apparent” extent of differentiation.

## DISCUSSION

### Image-based distinction between phases of early MSC differentiation

We have demonstrated for the first time that changes in nuclear architecture begin much earlier than previously described using various markers. This provides an effective method of determining the extent of early adipogenic differentiation of individual MSCs. Nuclear structures that are labeled with Hoechst 33342 are associated with different densities of DNA and nuclear bodies within the nucleus (Spagnol and Dahl, 2016; Efremov *et al.*, 2020). We confirm with nuclear rheology that a reduction in chromatin fluidity is also associated with early MSC differentiation. However, we were unable to show quantitative variation of common heterochromatin marker H3K9me3 and only slight changes at 72-h adipo using H3K27me3. Thus, there is some structural nuclear factor or collection of factors that changes in early MSC nuclei as early as 6–12 h after initiation of adipocytic differentiation.

### Toward an image-only method for cell differentiation

Machine learning models are able to capture transitions between groups of images that are not visible to the human eye. In this study, we labeled fixed cells and labeled with the DNA-intercalating dye Hoechst 33342. Obviously, a move toward in situ characterization of stemness and of differentiation requires that no fluorescent dyes be used: imaging fluorescent dyes produces oxidative radicals, and DNA-binding dyes in live cells can inhibit DNA transcription and translation as well as proper chromosomal segregation during adipogenesis. Recent advancements in phase contrast, differential interference contrast, Raman and light scattering microscopy (Lu *et al.*, 2015; Subedi *et al.*, 2020; Hsiao *et al.*, 2022) also show the ability to capture distinct subnuclear structures. Thus, fluorescence imaging may not be required. We suggest the possibility for MSCs being expanded many times over in flasks could be automatically imaged with a form of light microscopy, and from these images machine learning could be used to check for loss of “stemness.” Similarly, differentiation of cells could be tracked with imaging days to weeks before an extracellular matrix is produced. This diagnostic tool for MSCs could vastly expand their use in stem cell therapies and in tissue-specific engineering since the homogeneity of a culture could be determined without expensive processing.

## MATERIALS AND METHODS

### Cell culture

Human bone marrow–derived mesenchymal stem cells (hBM-MSCs) (RoosterBio, KT-002) were cultured in RoosterNourish-MSC medium traditional bioprocess medium (RoosterBio, KT-001) supplemented with 0.5% penicillin-streptomycin (Life Technologies). hBM-MSCs were cultured in an incubator with 5% CO_2_ and at 37°C. hBM-MSCs were subcultured with 0.25% trypsin-Ethylenediaminetetraacetic acid (EDTA) and subsequently passed every 3 or 4 d. hBM-MSCs at passage numbers within eight were used for further experiments. Adipogenic differentiation of hBM-MSCs were performed in glass-bottom 4-well chamber slides (Thermo Fisher Scientific) using StemPro Adipogenesis Differentiation Kit (Thermo Fisher Scientific) supplemented with 0.5% penicillin-streptomycin (Life Technologies).

### Immunofluorescence

hBM-MSCs were cultured in glass-bottom 4-well chamber slides (Thermo Fisher Scientific) with adipogenic differentiation media for different time intervals (0, 6/14, 24, 48, 72 h). Cells were fixed at room temperature with 4% paraformaldehyde for 15 min and then permeabilized with 0.25% Triton X-100 for 15 min. Three datasets were collected, all at 20x magnification (0.4 NA) on a widefield DMI6000 (Leica). Laser power, detector gain, and exposure time were the same for all images in a dataset (with the exception of the 6 h timepoint for anti-H3K27me3 which had a longer exposure, see below). In all datasets, cell nuclei were stained with 10 µg ml^−1^ Hoechst 33342 for 30 min in PBS at room temperature. For the first dataset, lipid droplets were stained with Nile Red (Thermo Fisher Scientific) at a final concentration of 1 μg ml^−1^ for 30 min at room temperature and F-actin was labeled with Rhodamine Phalloidin (Thermo Fisher Scientific) at a final concentration at 0.165 nM for 30 min at room temperature. For the second dataset, cells were incubated with mouse anti-H3K9me3 primary antibodies (Abcam, ab184677) at 1:1000 in 2.5% BSA overnight at 4°C. After three times washing with PBS, rabbit anti-mouse secondary antibodies conjugated with Alexa Fluor 488 (Thermo Fisher Scientific) were diluted in 2.5% BSA at 1:1000 and incubated on the cell slide for 1 h at room temperature. For the third dataset, the same procedure was used except mouse anti-H3K27me3 primary antibody (Abcam, ab6002) was used in place of H3K9me3 primary antibody, and F-actin was labeled with Rhodamine Phalloidin as above. Ten fields per differentiation timepoint were imaged for the Nile red dataset, and 50 or 51 fields per timepoint for the other two datasets. For the third dataset (which was used for the deep learning analysis), the numbers of cells per timepoint were 403, 437, 462, 271, and 391 for 0, 6, 24, 48 and 72 h, respectively.

### Particle tracking chromatin mechanics

DNA-binding probe telomeric repeat-binding factor 1 (TRF1) tagged mCherry2 was expressed in hBM-MSC via lentiviral transduction (SBI, LV500A-1). Hoechst 33342 (Thermo Fisher Scientific) was used to stain cell nuclei. Undifferentiated and 24 h adipogenic differentiated hBM-MSC were imaged at 3-minute intervals for 1 h by a spinning disk (CSU-X1 Yokogawa) confocal microscope (Nikon Eclipse Ti2) with an electron multiplier charge-coupled device camera (ANDOR iXon Life EMCCD). Our previous studies have described a particle-tracking technology where chromatin mobility was assessed by tracking chromatin-binding fluorescent probes. Briefly, the collected images were processed to remove the rotation or shift of nuclei; only the trajectories of fluorescent particles inside cell nuclei were tracked and analyzed. For each, the CD was calculated according to CD(τ) = <(X_t+τ_ − X_t_)^2^+(Y_t+τ_ − Y_t_)^2^>, where X_t_ and Y_t_ are the coordinates of the particle at timepoint t, and X_t+τ_ and Y_t+τ_ are the coordinates of the particle after lag time τ. We have previously shown that the CD of particles can be fit to a power law equation CD = D_eff_τ^β^, where D_eff_ represents the chromatin fluidity, β represents the energetic generation (Spagnol and Dahl, 2014; Armiger *et al.*, 2018; Tedesco *et al.*, 2022). CD of mCherry2-TRF1 was calculated versus lag time, τ, and fit to a power law. From this, the chromatin fluidity is determined as the prefactor we have also referred to as effective diffusivity (Ashwin *et al.*, 2020).

### Heterochromatin fluorescence quantitation

Images from the second and third dataset were used to measure average fluorescent intensity per timepoint. All the images of each heterochromatin marker (H3K9me3 or H3K27me3) were displayed with identical minimum and maximum display values using Fiji ImageJ. Then, cell nuclei were manually selected as regions of interest (ROIs), and the fluorescent intensity within ROIs was quantified using the “Measure” tool in Fiji ImageJ. The level of heterochromatin markers H3K9me3 and H3K27me3 was represented by the mean fluorescent intensity over 62 cells chosen arbitrarily from the available images.

### Image processing and CNN classification

#### Dataset.

**FIGURE 5:**
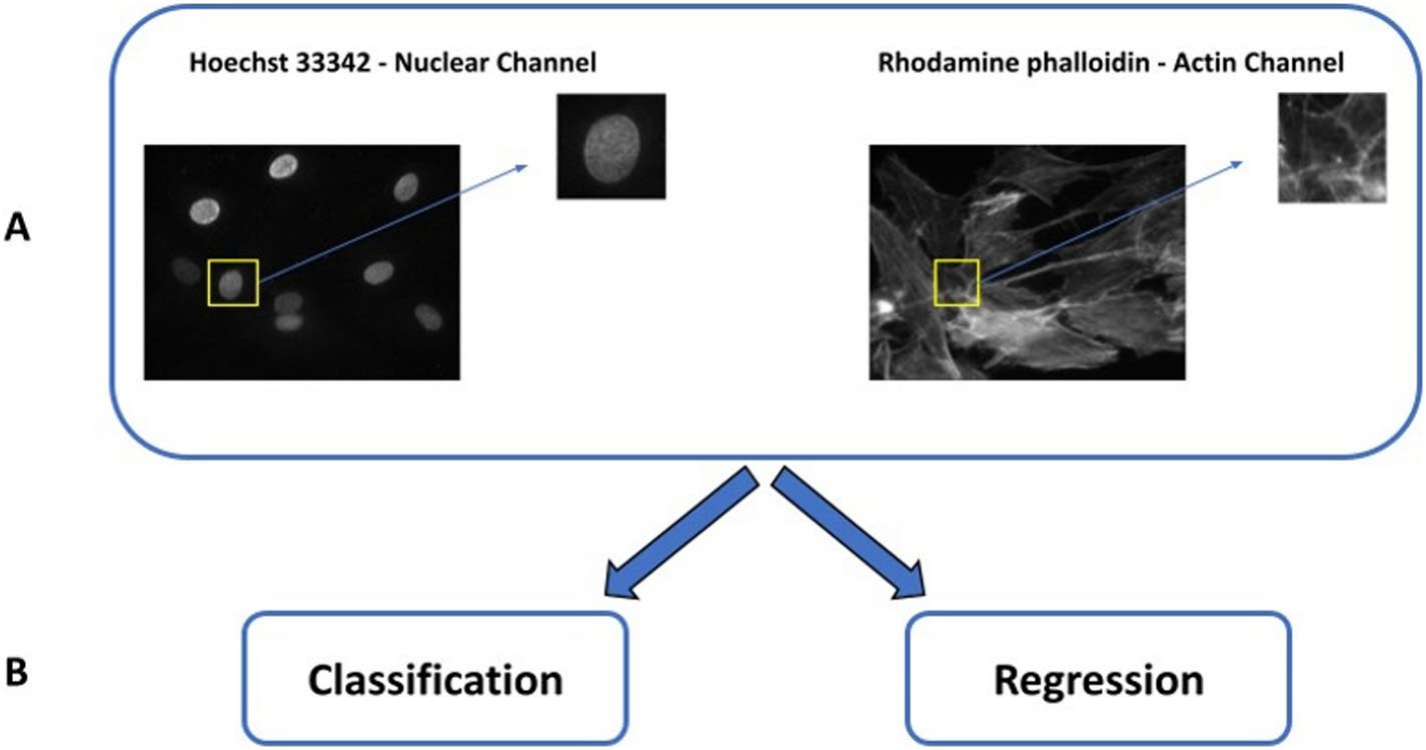
Machine learning pipeline for predicting and characterizing differentiation time periods of mesenchymal stem cells. (A) Input F-actin and chromatin images are segmented into regions containing individual nuclei, matched with the corresponding F-actin region. Datasets containing nuclei only, actin only, and combined nuclei and actin images are created Brightfield dataset is also created with the same region as the nuclei. (B) All datasets are processed with a set of CNN approaches.

The third dataset was used to train and evaluate deep learning models. The four channels were converted from 16 bit to 8 bit via a min-max scaler for each image; this corrects for the fact that the exposure time for the 6 h timepoint was shorter (70 s) than that for the other timepoints (85 s). The pixel size was 0.293 µm. For simultaneous analysis of Hoechst 33342–stained nucleus and phalloidin-stained actin, the frames of the nucleus and actin were combined into a single, two-channel image (Figure 5A).

#### Segmentation.

Individual cells were segmented using the Hoechst 33342 channel image by Otsu thresholding. To remove partial or incorrectly segmented cells, cells with heights and widths larger than 100 pixels or lower than 50 were removed. The maximum nuclear size (width and height) across all remaining cells was determined and used to define a rectangular frame centered around each single cell. These frames were used to cut out the corresponding regions of each channel.

#### Augmentation.

As each cell has different arbitrary orientations in a microscope image, the dataset was expanded (augmented) to reduce the impact of orientation and increase the number of images available for training and testing. This was done by adding copies of each image with rotations of 90, 180, and 270 degrees. After augmentation, the images were shuffled to generate diverse batches for each epoch of loss optimization.

#### Deep learning for classification and regression.

A simple CNN with four layers (one input, two hidden, and one output layer; Kingma and Welling, 2019). was trained for 25 epochs using two different output layers (Figure 5B). The regression model used a single output with linear activation, whereas the classification model used a feature/class vector of length 5 with softmax activation. A second model, MobileNet (Howard *et al.*, 2017), was used to compare the performance of the shallow CNN. The loss functions were mean squared error for regression and categorical cross-entropy for classification. All results reported are averages from 100 different random divisions of the images into training and test folds. All segmented and/or augmented images were kept in the same training or test fold as the original image.

### Software and data availability

The software used in this study is available at https://github.com/HoffmanOfGryffindor/msc-characterization. The images are available at https://doi.org/10.1184/R1/25718787.

### Funding

This work was supported in part by grant P41 GM103712 from the National Institute of General Medical Sciences.

## Abbreviations used:

CD: chromatin displacement
CNN: convolutional neural network
DIC: differential interference contrast
EDTA: ethylenediaminetetraacetic acid
GvHD: graft versus host disease
hBM-MSCs: human bone marrow-derived mesenchymal stem cells
*MSCs*: *mesenchymal stem cells*
PBS: phosphate buffered saline
ROIs: regions of interest
SSEA: stage-specific embryonic antigen
TRF1: telomeric repeat-binding factor 1
